# Knowledge of HIV infection and other sexually transmitted diseases among men who have sex with men in Finland

**DOI:** 10.1186/s12879-017-2203-5

**Published:** 2017-02-06

**Authors:** Tarja Suominen, Teppo Heikkinen, Marja Pakarinen, Anne-Mari Sepponen, Jari Kylmä

**Affiliations:** 1 University of Tampere, School of Health Sciences, Tampere, Finland; 2 Finnish AIDS Council, Tampere, Finland

**Keywords:** Knowledge, HIV, Transmission, PEP, MSM, Sexually transmitted diseases

## Abstract

**Background:**

The purpose of this study was to describe what is known about HIV infection and other sexually transmitted diseases, infection transmission routes, care, and sources of information, from the viewpoint of men having sex with men.

**Methods:**

National data (*n* = 2,072) was collected from June to August 2010 in Finland as part of a joint internet-based survey conducted in 38 countries (EMIS, European MSM Internet Sex Survey).

**Results:**

The respondents’ age, place of residence, highest education and employment status were statistically significantly related to how often the respondent sought information on HIV, testing and treatments, and what they knew about infection transmission routes. The respondents’ information seeking behavior was not seen as active regarding HIV infection and other sexually transmitted diseases.

**Conclusions:**

We should also consider the possibility of using internet-based interventions, especially in smaller and northern catchment areas, in order to improve the knowledge level of men having sex with men.

## Background

Finland is a country with about 5.5 million inhabitants, and to date (20.3.2016), 3,559 people have received an HIV diagnosis. Of all of the cases which noted the transmission route (84%), 72% were sexually transmitted. Of these, 32% were a result of sex between men [1]. The incidence of HIV infection in men engaging in sex with men is several times higher than seen in the population on average [1], and according to research conducted in 2006, the incidence of HIV in this group was 4.6% [2]. There has however been an increase in the number of HIV infections transmitted via sex between men in several European countries [3], and various methods have been developed to ascertain the real numbers of HIV infections in the European area [4]. The increase in infections connected to sex between men accelerated in Finland at the beginning of the 2000s, reaching a peak in 2007 when 72 new infections were diagnosed. Since this time, approximately 40 infections have been diagnosed each year [5].

A significant increase in the number of sexually transmitted diseases has been observed in the countries of Western Europe [6]. There have also been reports of a high prevalence of sexually transmitted diseases, especially among men engaging in sex with men (e.g. [7]).

In Finland, the most common sexually transmitted diseases are chlamydia, genital warts and genital herpes. In recent years the incidence of chlamydia has remained high [5]. About 244/100 000 new cases per year have been found 2012 to 2014 [1]. In 2014, the new cases of gonorrhea amounted to 286, of which 73% occurred in men. In 2014 in Finland, 203 cases of syphilis were diagnosed, of which 64% occurred in men [1]. This represented an increase of more than 50 cases compared to the previous year. The number of male syphilis infections has been increasing since 2006 and a considerable proportion of these cases have been due to sexual contact between men [5].

Factors conducive to risky behavior can be attributed to seeking sexual partners over the internet, an optimism as regards to the availability of effective medication for HIV, the burden of taking precautions against HIV, and the use of intoxicants [7]. There is also a connection between the availability of effective medication for HIV, the young age of men engaging in risky sexual behavior, and the increase in sexually transmitted diseases among men engaging in sex with men [8].

According to research, there is variation in what men engaging in sex with men know about HIV infection, HIV transmission and AIDS. This variation is also seen between studies, and whilst some studies have demonstrated a low level of knowledge [9], others report a good level of knowledge [10]. Even though people may be well informed about HIV infections, less may be known about its associated diseases. For example, Phillips et al. [11] noted that 75% of respondents in their study identifying themselves as homosexual or bisexual had heard of Kaposi’s Sarcoma, but only 6% knew what caused it.

Although many men who have sex with men know about HIV infection and its symptoms, they are not generally so well informed about other sexually transmitted diseases [7, 12]. For example, it has been observed that men having sex with men know relatively little about syphilis [13]. Even if their knowledge level of HIV is good, as many as 44% of this group may be unaware of their own HIV-positive status. This is especially noted in young men aged 18–29 [14]. However, there is not always a connection between the level of knowledge and the incidence of sexually transmitted diseases or HIV. Among those considering HIV infection to be a serious illness, it has been shown that the incidence of sexually transmitted diseases was less common [7].

In sex between men, the greatest risk for HIV infection and other sexually transmitted diseases is connected to unprotected anal intercourse. In anal intercourse, the best protection against HIV infection and other sexually transmitted diseases is the use of a condom and the correct type of lubricant cream. However, since not everyone uses a condom [15, 16] or when risk situations emerge for other reasons, then various post-exposure prophylactics (PEP) have been developed to prevent infection [17] and also pre-exposure prophylactics (PrEP). According to a study by Sidat et al. [18], there was no connection between an awareness of PEP medication and engaging in unprotected anal sex.

Earlier studies have reported a connection between a higher level of awareness regarding HIV, and variables such as having a high income level, seeking sexual partners on the internet, or having taken an HIV test more than one year earlier [19]; being HIV positive [11]; age (with youngest and oldest age groups having the least level of awareness) [19]; and having a high level of education [11, 20, 21]. On the other hand, in some studies income level, sexual orientation and age have not been observed as relating to the level of awareness. However, erroneous conceptions have been found about how HIV infection spreads, and include such vectors as insects, food, crockery and cutlery [21].

Sources of information on HIV infection most often include television, a sexual partner, information bulletins, and the internet [19]. The internet was particularly seen to be favored by those with the highest level of education and those who lived in a metropolis [22]. Wilkerson et al. [23] identified four types of information seekers: those who sought little information, those who sought only on the internet, those who primarily sought information from health care personnel, and those who used multiple sources. According to a study by Liu et al. [19], the highest level of knowledge related to HIV was among those who sought information through books, health care professionals, information bulletins and sexual partners. Among those who sought information from magazines and books, condom use was more common than in other groups, whereas among those who sought their information from health care professionals and public materials, condom use was found to be mediocre.

In a questionnaire and interview study (data collection 1997–1998) on Finnish homosexual and bisexual men, over half of the respondents reported that the media had the greatest effect on them engaging in safe sex [24]. The next most popular source of influence was the example of friends, followed by the homosexual community and the AIDS support centre [24].

The purpose of the study presented in this paper is to describe what is known among men having sex with men in Finland about HIV infections and other sexually transmitted diseases, what is known about how such infections spread and about treatments, and to identify the sources from which such information is obtained.

## Methods

The data for the study was gathered in June–August 2010, as part of the European MSM Internet Sex Survey (EMIS: [25]). The survey was a joint effort of 38 countries with a Europe-wide internet questionnaire in 25 different languages. Permission to conduct the study in each country was obtained from the Research Ethics Committee of the University of Portsmouth, United Kingdom (REC application number 08/09:21) [26, 27]. For the national recruitment in Finland national websites (*n* = 6), Planet Romeo, Manhut, Baydar, 7000 cards and 750 posters were used. The questionnaire was anonymous and relatively short. Participants were volunteers, response was by the respondent’s computer and individual respondents could not be identified. Each and every participant actively clicked a field to give informed consent. Respondents were adequately informed about both the practical implementation of the study and the publicizing of the results [26, 28].

Specific to the data collected in the Finnish component of the survey, the background variables elicited were the respondent’s age, place of residence, province of residence, duration of stay in Finland, highest educational qualification, employment status, HIV positivity, HIV status of permanent partner, occurrence of sexually transmitted diseases (yes/no), and whether they had ever been treated with a PEP medication. The information regarding HIV infections, tests and treatments was elicited with seven statements. The degree of the respondent’s knowledge about the spread of HIV infections was evaluated with six statements, and what they knew about PEP medication was evaluated with three statements. The statements were in five-step Likert form. For analysis, the statements were reclassified into three classes (‘within the last month’, ‘less often’, and ‘never’), or into two classes (‘I knew this already’/‘I am not sure’, and ‘I don’t understand’/‘I don’t believe this’. The approaches to finding information were assessed with four statements which offered five response options. For purpose of analysis, these responses were later classified into three classes (‘in the last month’, ‘less often’, ‘never’). The data was analyzed using SPSS software version 16.0. Connections between variables were examined with cross-tabulations and Pearson’s x^2^ test of independence. A five percent risk limit was set as the level of significance [28].

## Results

### Participants

The survey respondents in Finland numbered 2,072, and their average age was 34.3 years (SD = 11.5). 32% were resident in an area with population of less than 100,000, 30% in mid-sized areas, and 38% in an area with a population greater than 500,000. Half of the respondents (50.4%) lived in other provinces than the largest province of Uusimaa, situated in southern Finland. There were 196 responses to the question regarding the length of stay in Finland (intended for those not born in Finland), and half of these (50.5%) had lived in Finland for ≤ 15 years. About half (48.9%) of all the respondents had either a college-level qualification or higher, and just over half (53.8%) were working full-time.

Most of the respondents were HIV-negative, and 3.2% were HIV-positive. Of the respondents in a permanent relationship with a man, 87% (*n* = 867) had the same HIV status as their partner. Of those in a permanent relationship with a woman, 90% (*n* = 156) had the same HIV status as their partner. This status might either be positive or negative. Overall, 73.5% of respondents had never been diagnosed with a sexually transmitted disease. Relating to type, 0.5–8.7% of respondents had had some form of sexually transmitted disease: chlamydia (8.7%), genital warts (8.4%), gonorrhea (7.2%), herpes (3.1%), syphilis (2.8%) and hepatitis C (0.5%). Most of the respondents had not been treated with a PEP preventive medication (98.7%).

### Respondents’ awareness of HIV infection and sexually transmitted diseases

By and large, the respondents were well aware of HIV infection and sexually transmitted diseases, albeit that only about half of them knew that the effective treatment of HIV infection reduces the risk of passing it on (Table 1). Less than half of the respondents were aware that the risk of passing on an HIV infection is greater in sex between an HIV-positive and an HIV-negative man, if one or the other also has another form of sexually transmitted disease (Table 2). The statements ascertaining the respondents’ knowledge were correctly answered (Tables 1 and 2) by 18.3% (*n* = 370) of the total of Finnish responses. Of the respondents diagnosed as HIV-positive, 24.6% (*n* = 65) responded correctly to the knowledge statements, and of those who were diagnosed as HIV-negative, 19.8% (*n* = 1,227) responded correctly.

**Table 1:** What is known about HIV, tests and treatments

Did you know this about HIV, tests and treatments?	I already knew this %	I was not certain about this %	I did not yet know this %	I don’t understand this %	I don’t believe this %
AIDS is caused by a virus called HIV (*n* = 2066)	98.9	0.6	0.1	0.0	0.3
You cannot be confident about whether someone has HIV or not from their appearance (*n* = 2066)	96.7	1.4	0.3	0.1	1.5
There is a medical test that can show whether or not you have HIV (*n* = 2062)	98.7	0.7	0.1	0.1	0.3
If someone becomes infected with HIV it may take several weeks before it can be detected in a test (*n* = 2061)	93.4	5.0	1.6	0.0	0.1
There is currently no cure for HIV infection (*n* = 1399)	93.8	4.4	0.4	0.1	1.4
HIV infection can be controlled with medicines so that its impact on health is much less (*n* = 2065)	96.0	3.3	0.4	0.0	0.2
Effective treatment of HIV infection reduces the risk of HIV being transmitted (*n* = 2066)	54.3	21.9	13.9	1.3	8.6

**Table 2:** Knowledge of transmission

Did you know this about HIV and sexually transmitted infections	I already knew this %	I was not certain about this %	I did not yet know this %	I don’t understand this %	I don’t believe this %
HIV cannot be passed during kissing, including deep kissing, because saliva does not transmit HIV (*n* = 2059)	77.0	17.2	2.4	0.3	3.1
You can pick up HIV through your penis while being ‘active’ in unprotected anal or vaginal sex (fucking) with an infected partner, even if you don’t ejaculate (*n* = 2054)	93.0	5.6	0.9	0.0	0.4
You can pick up HIV through your rectum while being ‘passive’ in unprotected anal sex (being fucked) with an infected partner (*n* = 2058)	98.0	1.5	0.3	0.0	0.1
Even without ejaculation, oral sex (sucking and being sucked) carries a risk of infection with syphilis or gonorrhoea (*n* = 2059)	85.3	11.9	2.5	0.0	0.2
When HIV infected and uninfected men have sex together, the chances of HIV being passed on are greater if either partner has another sexually transmitted infection (*n* = 2055)	49.2	23.1	24.4	1.3	1.9
Most sexually transmitted infections can be passed on more easily than HIV (*n* = 2053)	77.8	14.3	6.0	0.4	1.5

One fifth (20%) of the respondents did not know that several other sexually transmitted diseases may be passed on more easily than HIV. Additionally, about 15% of respondents did not know that even without ejaculation, oral sex exposes participants to being infected with both syphilis and gonorrhea (Table 2).

Little was known about Post Exposure Prophylaxis (PEP). Pre Exposure Prophylaxis (PrEP) was not asked about, while not available at the time of the study. One in four (26.5%) knew that PEP attempts to stop HIV infection taking place after a person is exposed to the virus, A similar amount (25.4%) knew that PEP should be started as soon as possible after exposure, and around one in six (15.2%) knew that PEP is a one month course of anti-HIV drugs. Only 1.3% of respondents had been treated with PEP. Overall, 13.5% of the respondents (*n* = 279) responded correctly to the statements about PEP. Of the respondents who were diagnosed as HIV-positive (34: *n* = 65), just over half (52.3%) responded correctly to the PEP statements. The corresponding figure for HIV-negative respondents was 13.6% (167: *n* = 1,227).

There was a statistically significant effect corresponding to the size of the respondent’s area of residence, their age, highest qualification and employment status, on how often they sought information on HIV, tests and treatments, and what they knew about the spread of infection (Table 3). Especially, it was noted that those respondents who had had a sexually transmitted disease were statistically significantly better informed about HIV, tests, treatments, the spread of infection and PEP medication.

**Table 3:** Information seeking and knowledge

		Age			Place of residence			Highest education		Employment	
		Under 25	25-40	Over 40	Pop. Over 500 000	Pop. 100 000-499 999	Pop. Under 100 000	College level or higher	Others	Full time	Others
Information seeking	During last month	**2**	**13**	**9**	**15**	**2**	**6**	**16**	**8**	**18**	**6**
		**0.4%**	**1.3%**	**1.5%**	**2.0%**	**0.3%**	**0.9%**	**1.6%**	**0.8%**	**1.6%**	**0.6%**
	More seldom	**379**	**857**	**516**	**673**	**513**	**521**	**889**	**858**	**968**	**777**
		**78.8%**	**86.3%**	**87.8%**	**88.3%**	**85.9%**	**80.8%**	**88.5%**	**81.7%**	**87.6%**	**81.9%**
	Never	**100**	**123**	**63**	**74**	**82**	**118**	**99**	**184**	**119**	**166**
		**20.8%**	**12.4%**	**10.7%**	**9.7%**	**13.7%**	**18.3%**	**9.9%**	**17.5%**	**10.8%**	**17.5%**
Knowledge about HIV, tests and treatments	Already knowing	472	985	585	758	591	637	997	1035	**1101**	**932**
		97.5%	98.7%	99.2%	99.1%	98.8%	98.0%	99.1%	98.3%	**99.3%**	**98.0%**
	Not certain, know, understand, believe	12	13	5	7	7	13	9	18	**8**	**19**
		2.5%	1.3%	0.8%	0.9%	1.2%	2.0%	0.9%	1.7%	**0.7%**	**2.0%**
Knowledge about infection transmission	Already knowing	**446**	**951**	**562**	**741**	**567**	**599**	**971**	**983**	**1069**	**885**
		**92.1%**	**95.3%**	**95.3%**	**96.9%**	**94.8%**	**92.2%**	**96.5%**	**93.4%**	**96.4%**	**93.1%**
	Not certain, know, understand, believe	**38**	**47**	**28**	**24**	**31**	**51**	**35**	**70**	**40**	**66**
		**7.9%**	**4.7%**	**4.7%**	**3.1%**	**5.2%**	**7.8%**	**3.5%**	**6.6%**	**3.6%**	**6.9%**
Knowledge about PEP	Already knowing	**77**	**242**	**121**	**186**	**110**	**130**	**235**	**205**	249	190
		**15.9%**	**24.2%**	**20.5%**	**24.3%**	**18.4%**	**20.0%**	**23.4%**	**19.5%**	22.5%	20.0%
	Not certain, know, understand, believe	**407**	**756**	**469**	**579**	**488**	**520**	**771**	**848**	860	761
		**84.1%**	**75.8%**	**79.5%**	**75.7%**	**81.6%**	**80.0%**	**76.6%**	**80.5%**	77.5%	80.0%

***p*** **-value < 0.05**

*p*-value > 0.05

### Information seeking

Active information seeking about HIV infection and other sexually transmitted diseases was not found to be a common practice. Of those living in rural areas, one fifth had never sought information (Table 3). Seeking information through a telephone helpline was not a common practice, and about 9/10 had never sought telephone advice (Table 4). However, respondents who were diagnosed with a sexually transmitted disease were significantly more active in seeking information and using helplines.

**Table 4:** Information seeking

Information seeking about HIV and sexually transmitted infections (STI)	Within the last month %	Not so often %	Never %
When was the last time you saw or heard any information about HIV or STIs specifically for men who have sex with men? (*n* = 2058)	43.1	51.4	5.5
When was the last time you saw any information about HIV or STIs in a magazine or newspaper? (*n* = 2054)	31.0	62.1	6.9
When was the last time you actively looked for information about HIV or STIs on the internet? (*n* = 2048)	15.5	70.5	14.0
When did you last call a telephone helpline for information about HIV or STIs (*n* = 2061)	0.5	7.0	92.4

## Discussion

The instrument used in the study was developed in international co-operation among 38 countries as part of the EMIS Research Project [27], and its validity was extensively checked. The instrument was pre-tested in each country and the translation into Finnish was made using a double translation approach [28]. The data collection was internet-based which could be seen as a limitation, but it was advertised and delivered nationwide throughout Finland. Men living in Finland and engaging in sex with men were able to participate in the questionnaire, which was offered in 25 different languages.

By and large the respondents showed a good awareness of HIV infection and sexually transmitted diseases. This corresponds with the findings of earlier research data [10], but contradictory studies also report that little is known [9]. There have been earlier connections drawn between the respondents’ age and the size of their place of residence and, relating to those who live in cramped/shared accommodation but no extensive study has previously been carried out in Finland.

Little was known about Post Exposure Prophylaxis (PEP). Of those diagnosed as HIV-positive, 34 (52.3%: *n* = 65) responded correctly to all of the statements about PEP. It seems that the availability of PEP is not well known, however, ensuring that all MSM are aware of PEP is a challenging undertaking in rural areas. Furthermore, in some countries like Finland, PEP is not available free of charge after sexual exposure. Information seeking on HIV and other sexually transmitted diseases was also not found to be extensive among those without experience of such infections (See [19, 23]).

## Conclusions

Preventive information dissemination should be stepped up, as those respondents who did not have/had not had any infection did not know much about HIV infection and other sexually transmitted diseases, how they spread, or how they can be treated. Maximizing the opportunities afforded by the internet to improve the level of knowledge among men engaging in sex with men should be considered, while in some areas, like in eastern and northern parts of Finland the distances are long to meet health care professionals. This is particularly so in small areas that may not offer any widespread access to information, or have limited communities where such knowledge can be shared in person. The possibility of using internet-based interventions should especially be considered in smaller and northern catchment areas, and their impact evaluated in further studies.

## Abbreviations

AIDS: Acquired immune deficiency syndrome
EMIS: European MSM Internet Sex Survey
HIV: Human immunodeficiency virus
MSM: Men who have sex with men
PEP: Post exposure prophylaxis
PrEP: Pre-exposure prophylactics
SD: Standard deviation
STI: Sexually transmitted infection
